# Body surface area–adjusted median nerve cross-sectional area and multimodal ultrasound improve diagnosis of carpal tunnel syndrome

**DOI:** 10.3389/fsurg.2026.1774737

**Published:** 2026-02-23

**Authors:** Boyi Yu, Jie Du, Yansong Liu, Lili Zhang, Hongyu Li, Fangfang Sun, Lifang Liu, Chao Zhang, Xinyue Liu, Feng Hu, Linlin Shao, Mengqin Sun, Lirong Zhao

**Affiliations:** Ultrasound Diagnostic Center, The First Hospital Affiliated to Jilin University, Changchun, Jilin, China

**Keywords:** body surface area, carpal tunnel syndrome, high-frequency ultrasound, median nerve cross-sectional area, shear wave elastography, superb microvascular imaging, Z-score

## Abstract

**Background:**

To evaluate the diagnostic performance of high-frequency ultrasound combined with Superb Microvascular Imaging (SMI) and Shear Wave Elastography (SWE) for carpal tunnel syndrome (CTS), and to develop an individualized diagnostic approach using a body surface area (BSA)–adjusted median nerve CSA at the pisiform level.

**Materials and methods:**

This retrospective study included 47 wrists with carpal tunnel syndrome (CTS) and 94 control wrists. Median nerve cross-sectional area (CSA) was measured at four anatomical sites. Superb Microvascular Imaging (SMI) and Shear Wave Elastography (SWE) were used to assess intraneural vascularity and stiffness, respectively. A linear regression model was developed to estimate the expected CSA at the pisiform level based on body surface area (BSA), and a BSA-based Z-score was calculated accordingly. Receiver operating characteristic (ROC) analyses were performed to compare the diagnostic performance of (i) a fixed CSA cutoff at the pisiform level, (ii) the BSA-based Z-score, and (iii) a combined SMI + SWE logistic regression model.

**Results:**

Ultrasound parameters differed significantly between the CTS and control groups (*P* < 0.05). The BSA-based Z-score derived from the CSA at the pisiform level yielded an AUC of 0.924 (95% CI 0.879–0.969) and improved specificity (83%; 95% CI 0.738–0.899) compared with the fixed CSA cutoff (75%; 95% CI 0.644–0.829). In multivariable analysis, SMI- and SWE-derived parameters remained independent predictors of CTS (*P* < 0.001). The combined SMI + SWE logistic regression model demonstrated the best diagnostic performance (AUC 0.944; 95% CI 0.906–0.982), with 83% sensitivity (95% CI 0.692–0.924) and 90% specificity (95% CI 0.826–0.955).

**Conclusion:**

High-frequency ultrasound combined with Superb Microvascular Imaging (SMI) and Shear Wave Elastography (SWE) enables accurate, noninvasive evaluation of CTS. A BSA-based CSA Z-score improves specificity in CSA-based diagnosis, and integrating SMI and SWE further enhances overall diagnostic performance.

## 1 Introduction

Carpal tunnel syndrome (CTS) is a common peripheral entrapment neuropathy caused by elevated pressure within the carpal tunnel and consequent compression of the median nerve (MN). It typically presents with pain, numbness, and sensory disturbance in the MN distribution and may progress to weakness and thenar atrophy in severe cases, substantially impairing daily activities and work performance (1–4).

Diagnosis currently relies on an integrated assessment of clinical history, physical examination, and electrophysiological testing (EDX) (5). Although EDX is widely regarded as the reference standard, its performance may be affected by patient cooperation and operator or equipment factors, it can be uncomfortable, and it does not directly visualize MN morphology or localize the site of compression; sensitivity may also be limited in early or mild CTS (6–8). High-frequency ultrasound provides real-time, high-resolution visualization of the MN and surrounding structures, helps identify potential compressive factors, and depicts morphologic changes that can support diagnosis and treatment planning (9).

Conventional ultrasound assessment incorporates parameters such as median nerve cross-sectional area (CSA), flattening ratio, and transverse carpal ligament thickness, while emerging techniques including Shear Wave Elastography (SWE) and Superb Microvascular Imaging (SMI) provide complementary information on nerve stiffness and intraneural microvascularity (10, 11). Among these, CSA at the carpal tunnel inlet (pisiform level) is the most widely used metric (12); however, reported upper limits of normal vary widely (13–21) and substantial inter-individual variability has been described (22–25), limiting the applicability of a single fixed cutoff. Because body size may influence CSA, we aimed to establish a body surface area (BSA)-based regression equation to derive individualized reference values and corresponding Z-scores for CTS diagnosis, and to compare this approach with conventional fixed thresholds [e.g., 0.11 cm^2^ (11 mm^2^)]. In addition, we evaluated the diagnostic performance of two-dimensional ultrasound, SMI, SWE, and an SMI + SWE combined model to refine ultrasound-based diagnostic criteria and improve clinical decision-making.

## 2 Materials and methods

### 2.1 Ethics approval and consent

This study was approved by the Institutional Review Board of the First Hospital of Jilin University. Written informed consent to participate in this study was obtained from all participants prior to enrollment. The study was conducted in accordance with the Declaration of Helsinki.

### 2.2 Participants

Between May 2024 and May 2025, we retrospectively identified 32 patients with carpal tunnel syndrome (CTS) from the Department of Hand and Foot Surgery, Lequn Campus, First Hospital of Jilin University. In total, 47 wrists were included (8 wrists from 5 men and 39 wrists from 27 women; age, 42–74 years). Fifteen patients had bilateral CTS and 17 had unilateral CTS. CTS was diagnosed based on typical clinical symptoms and electrophysiological examinations. Clinical manifestations included unilateral or bilateral numbness in the median nerve distribution (thumb to the radial half of the ring finger), weakness, wrist discomfort, and nocturnal paresthesia. Physical examination demonstrated positive Tinel's sign and Phalen's test on the affected side; thenar atrophy was noted in advanced cases. All CTS patients underwent electrophysiological examinations.

The control group comprised 94 volunteers recruited from individuals undergoing routine physical examinations at the Ultrasound Clinic, Lequn Campus, First Hospital of Jilin University during the same period. One wrist was randomly selected from each participant (20 wrists from 20 men and 74 wrists from 74 women; age, 39–75 years). Controls had no symptoms suggestive of CTS.

Exclusion criteria for both groups were: (1) age <18 years; (2) history of wrist trauma or surgery; (3) anatomical variants, including a persistent median artery or bifid median nerve; (4) systemic diseases (e.g., diabetes mellitus, gout, acromegaly, rheumatoid arthritis, or hyperparathyroidism); (5) history or clinical evidence of polyneuropathy or hereditary neuropathy with liability to pressure palsies; (6) other neurological disorders such as cervical radiculopathy or brachial plexus injury; and (7) incomplete clinical data or inability to cooperate with the examination procedures.

### 2.3 Ultrasound Equipment

All ultrasound examinations were performed using a Canon Aplio i800 system (Canon Medical Systems, Japan) with i24LX8 and i18LX5 linear-array transducers, which support Superb Microvascular Imaging (SMI) and Shear Wave Elastography (SWE). Musculoskeletal/nerve presets were used, and imaging settings were adjusted to optimize visualization of the median nerve and maintained as consistently as possible across participants.

### 2.4 Ultrasound Examination and Data Acquisition

Participants were seated comfortably with the forearm resting on the examination table, the palm facing upward, fingers slightly flexed, and the wrist in a relaxed neutral position. The transducer was first aligned with the longitudinal axis of the median nerve to identify the nerve course and key landmarks; cross-sectional images were then obtained by rotating the probe to the transverse plane. Throughout scanning, the probe was kept perpendicular to the skin to minimize obliquity, and excessive pressure was avoided to prevent artificial deformation of the nerve. Adequate coupling gel was applied to ensure optimal acoustic contact.

The sonographer who performed the ultrasound examinations (10 years of experience in musculoskeletal ultrasound) was blinded to the electrophysiological results at the time of image acquisition. Measurements were performed using a standardized protocol, and quantitative parameters were recorded at the time of examination.

### 2.5 Two-dimensional (2D) ultrasound

**Figure 1:**
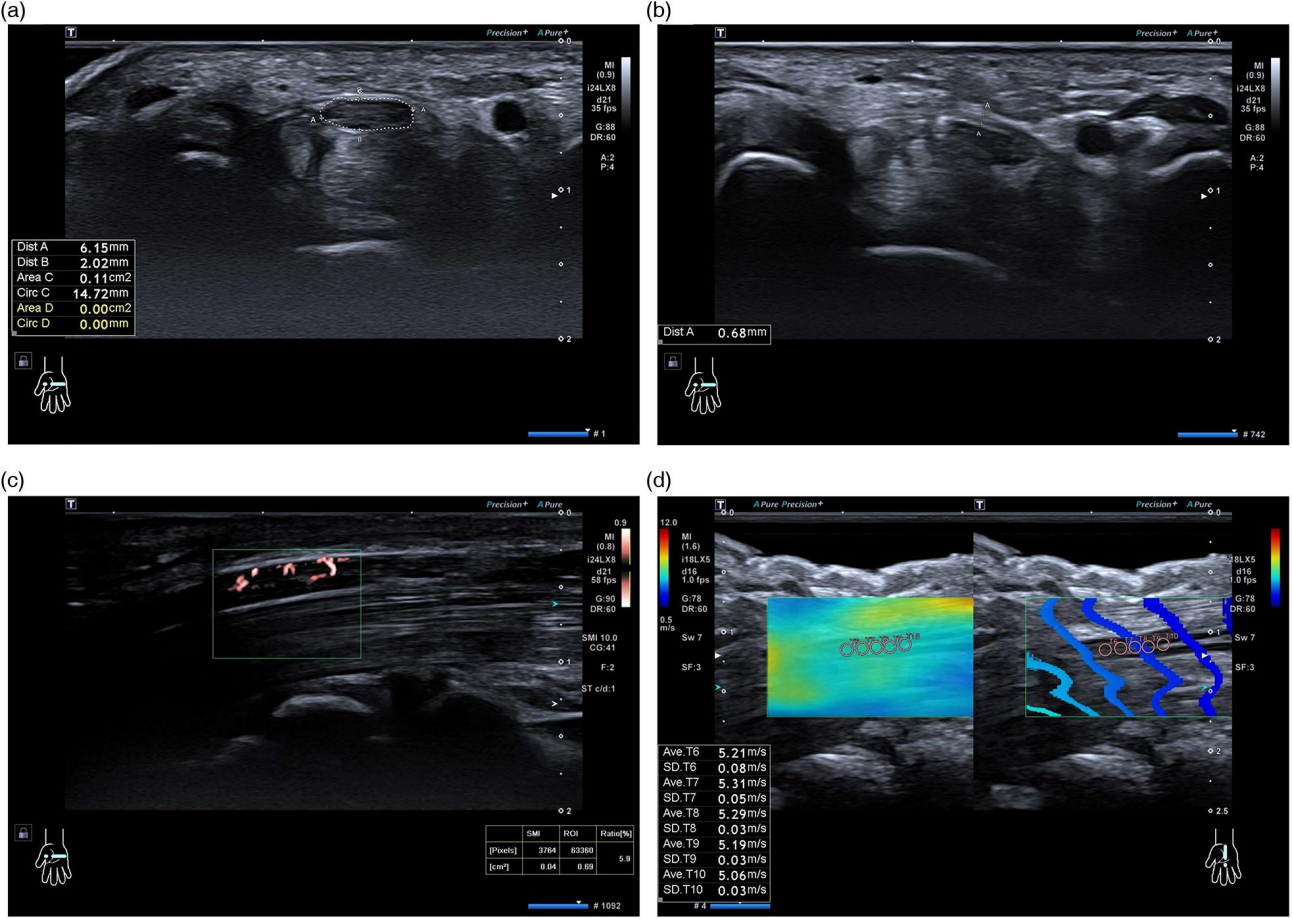
Ultrasound measurement of the median nerve using B-mode, SMI, and SWE techniques. **(a)** Cross-sectional area (CSA) of the median nerve measured on B-mode ultrasound at the pisiform level. **(b)** Transverse carpal ligament (TCL) thickness measurement anterior to the median nerve. **(c)** Superb Microvascular Imaging (SMI) showing intraneural blood flow signals. **(d)** Shear Wave Elastography (SWE) image displaying a 1-mm region of interest (ROI) within the median nerve.

The median nerve was systematically scanned from the distal arm to the wrist to evaluate morphology, course, and surrounding tissue abnormalities. On transverse images, the median nerve cross-sectional area (CSA) was measured at four predefined levels: the pisiform level (carpal tunnel inlet), the hook of hamate level (carpal tunnel outlet), the distal wrist crease, and the pronator quadratus level (distal forearm). CSA was obtained by manual tracing along the inner margin of the hyperechoic epineurial rim (Figure 1a). At each level, the flattening ratio (FR) was calculated as the transverse diameter divided by the anteroposterior diameter. *Δ*CSA was defined as the difference in CSA between the pisiform and pronator quadratus levels. To improve delineation of the transverse carpal ligament (TCL), participants were asked to gently move the fingertips to help distinguish the TCL from the palmar superficial fascia; TCL thickness was measured directly superficial to the median nerve (Figure 1b). Each measurement was repeated three times by the same examiner, and the mean value was used for analysis. The BSA-based Z-score model was derived from CSA measured at the pisiform level.

### 2.6 Superb Microvascular Imaging (SMI)

A 24-MHz high-frequency linear transducer was gently placed on the wrist with sufficient coupling gel to avoid excessive compression. After obtaining routine transverse 2D images at the pisiform and hook of hamate levels, the probe was rotated by 90° to obtain a longitudinal (long-axis) view of the median nerve and SMI was activated. The region of interest (sampling frame) was centered on the median nerve, and the frame size, position, and flow velocity scale were set identically for all participants and kept constant during acquisition. Intraneural flow was quantified automatically as the pixel ratio, defined as the percentage of color pixels within the sampling frame relative to the total pixel count. Care was taken to avoid contamination from adjacent non-neural vascular signals (Figure 1c). Measurements were repeated twice, and the mean value was used for analysis.

### 2.7 Shear Wave Elastography (SWE)

Shear wave elastography was performed using an 18-MHz linear transducer with minimal probe pressure. The SWE display scale was set to 0.5–12 m/s. Images were acquired only when the shear wave propagation lines were smooth and parallel. A 1-mm circular region of interest (ROI) was placed within the median nerve, avoiding the hyperechoic epineurium and adjacent structures. For each acquisition, five consecutive measurements with stable propagation and without obvious artifacts were recorded (Figure 1d), and their mean value was calculated. The acquisition was repeated twice, and the average of the two mean values was used for analysis. All ultrasound settings were kept consistent, and all examinations were performed by the same experienced operator.

### 2.8 General Data Collection

Demographic and anthropometric variables were collected, including sex, age, height, weight, wrist circumference, body mass index (BMI), and body surface area (BSA). Height and weight were measured using standardized equipment with participants standing upright barefoot and without heavy clothing. BMI was calculated as weight (kg)/height^2^ (m^2^). BSA was calculated using the Du Bois formula. Wrist circumference was measured circumferentially at the distal wrist crease using a flexible tape measure with the wrist in a relaxed neutral position. All measurements were standardized and obtained by a single examiner to ensure data accuracy and consistency.

### 2.9 Calculation of Z-scores

Individualized *Z*-scores adjusted by BSA were calculated using the formula: *Z* *=* *(X−µ)/σ* *=* *(measured CSA−BSA-predicted CSA)/standard deviation*. *X* represented the mean of three actual CSA measurements, *µ* represented the predicted CSA based on BSA, and *σ* was the standard deviation. Thus, the formula can be expressed as: *Z-score × σ* *=* *measured CSA−predicted CSA*, enabling calculation of individualized upper limits of normal (ULN) CSA values based on BSA.

### 2.10 Statistical analysis

All statistical analyses were performed using SPSS Statistics 29.0 (IBM, Armonk, NY, USA). Normality was assessed using the Shapiro–Wilk test. Continuous variables were compared between groups using the independent-samples *t*-test for normally distributed data or the Mann–Whitney *U* test for non-normally distributed data. Categorical variables were compared using the chi-square test. Normally distributed data are presented as mean ± standard deviation (SD), and non-normally distributed data as median [interquartile range (IQR)]. Spearman's rank correlation was used for correlation analyses when variables were not normally distributed. McNemar's test was used for paired categorical comparisons when applicable. Linear regression analysis was performed to establish a BSA-based prediction equation for median nerve CSA at the pisiform level and to derive individualized Z-scores. Receiver operating characteristic (ROC) analyses were conducted to evaluate the diagnostic performance of 2D ultrasound parameters, SMI, SWE, the BSA-based Z-score, and the combined SMI + SWE logistic regression model. Areas under the ROC curve (AUCs), optimal cutoff values (determined by the Youden index), sensitivity, and specificity were calculated. AUCs were reported with 95% confidence intervals (CIs) from ROC analyses. In addition, 95% CIs for sensitivity and specificity at the selected cutoffs were calculated using exact binomial (Clopper–Pearson) methods. Logistic regression analysis was performed with CTS status as the dependent variable and SMI and SWE parameters as independent variables. In addition, a sensitivity analysis was performed by further adjusting the multivariable logistic regression model for age and sex to assess the robustness of the associations. A two-sided *P* value < 0.05 was considered statistically significant.

## 3 Results

### 3.1 General information

A total of 126 participants (141 wrists) were included in the analysis. The CTS group comprised 32 patients (47 wrists; 8 wrists from 5 men and 39 wrists from 27 women), and the control group comprised 94 healthy subjects (94 wrists; 20 wrists from 20 men and 74 wrists from 74 women). The mean age was 53.98 ± 8.63 years in the CTS group and 53.52 ± 8.28 years in the control group (*P* = 0.86). There were no significant between-group differences in sex distribution, body weight, or body surface area (BSA) (all *P* > 0.05). In contrast, the CTS group had a lower height (158.85 ± 9.92 vs. 163.11 ± 9.68 cm, *P* = 0.02), a higher BMI (27.00 ± 5.16 vs. 24.95 ± 3.94 kg/m^2^, *P* = 0.04), and a larger wrist circumference (16.58 ± 1.37 vs. 16.02 ± 1.45 cm, *P* = 0.03) (Table 1).

**Table 1:** Comparison of baseline characteristics between the CTS group and the control group.

Characteristics	Total (*n* = 141)	CTS group (*n* = 47)	Control group (*n* = 94)	*P* value
Sex (male/female), *n* (%)	28 (19.9%)/113 (80.1%)	8 (17.0%)/39 (83.0%)	20 (21.3%)/74 (78.7%)	0.55
Age (years)	53.67 ± 8.37	53.98 ± 8.63	53.52 ± 8.28	0.86
Height (cm)	161.69 ± 9.93	158.85 ± 9.92	163.11 ± 9.68	0.02*
Weight (kg)	67.29 ± 14.85	68.36 ± 16.08	66.75 ± 14.26	0.78
BMI(kg/m^2^)	25.64 ± 4.48	27.00 ± 5.16	24.95 ± 3.94	0.04*
BSA(m^2^)	1.71 ± 0.21	1.70 ± 0.21	1.72 ± 0.21	0.46
Wrist circumference (cm)	16.21 ± 1.44	16.58 ± 1.37	16.02 ± 1.45	0.03*

Data are presented as mean ± standard deviation (SD) or number (percentage), as appropriate.

BMI, body mass index; BSA, body surface area.

* *P* < 0.05 indicates statistical significance.

### 3.2 Comparison of ultrasound parameters between the CTS and control groups

Two-dimensional (2D) ultrasound parameters, Superb Microvascular Imaging pixel ratio (SMI PR), and Shear Wave Elastography (SWE)–derived metrics are summarized in Table 2. Compared with controls, the CTS group showed significantly higher CSA at the pisiform (CSA1), hook of hamate (CSA2), and distal wrist crease (CSA3) levels, a higher flattening ratio at the hook of hamate level (FR2), a larger *Δ*CSA, and increased transverse carpal ligament (TCL) thickness (all *P* < 0.05). In addition, SMI PR and SWE parameters [elastic modulus [E] and shear wave velocity [SWV]] were significantly higher in the CTS group than in the control group (all *P* < 0.05). No significant between-group differences were observed for FR1, FR3, CSA4, or FR4 (all *P* > 0.05).

**Table 2:** Comparison of ultrasound parameters between the CTS group and the control group.

Parameters	CTS group (*n* = 47)	Control group (*n* = 94)	*P* value
CSA1 (cm^2^)	0.16 ± 0.05	0.10 ± 0.02	<0.001*
FR1	3.11 ± 0.93	2.98 ± 0.62	0.86
CSA2 (cm^2^)	0.10 ± 0.02	0.09 ± 0.01	0.001*
FR2	4.32 ± 0.79	3.69 ± 0.71	<0.001*
CSA3 (cm^2^)	0.14 ± 0.05	0.09 ± 0.02	<0.001*
FR3	2.72 ± 0.68	2.83 ± 0.76	0.38
CSA4 (cm^2^)	0.07 ± 0.01	0.07 ± 0.02	0.25
FR4	1.80 ± 0.45	1.82 ± 0.49	0.95
*Δ*CSA (cm^2^)	0.09 ± 0.05	0.03 ± 0.02	<0.001*
TCL (mm)	0.93 ± 0.11	0.69 ± 0.11	<0.001*
SMI PR (%)	3.50 ± 1.79	0.94 ± 0.82	<0.001*
E (kPa)	115.91 ± 16.76	86.00 ± 20.13	<0.001*
SWV (m/s)	6.18 ± 0.45	5.29 ± 0.65	<0.001*

Data are presented as mean ± standard deviation (SD).

CSA1–CSA4, median nerve cross-sectional area measured at the pisiform (carpal tunnel inlet), hook of hamate (carpal tunnel outlet), distal wrist crease, and pronator quadratus levels, respectively. FR1–FR4, flattening ratio measured at the corresponding levels. *Δ*CSA, difference in CSA between the pisiform and pronator quadratus levels; TCL, transverse carpal ligament thickness; SMI PR, superb microvascular imaging pixel ratio; E, elastic modulus; SWV, shear wave velocity.

* *P* < 0.05 indicates statistical significance.

### 3.3 ROC curve analysis of ultrasound parameters in the CTS group

**Figure 2:**
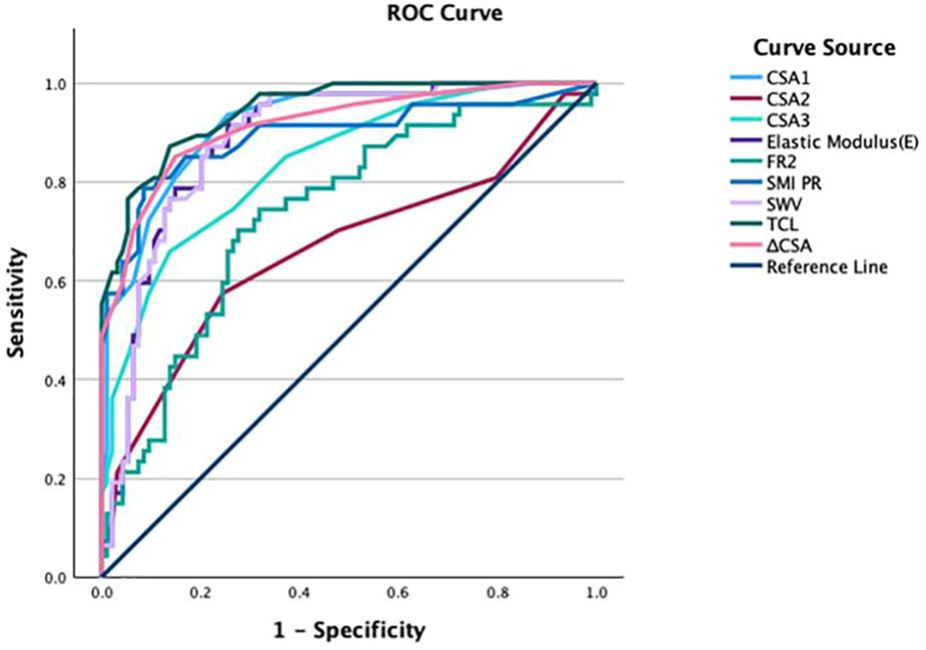
ROC curves of ultrasound parameters for CTS diagnosis.

Receiver operating characteristic (ROC) analyses were performed to evaluate the ability of each ultrasound parameter to differentiate CTS wrists from control wrists (Figure 2). Table 3 summarizes the area under the ROC curve (AUC), optimal cutoff values, sensitivity, and specificity for each parameter. For each parameter, AUCs as well as sensitivities and specificities are reported with 95% confidence intervals (Table 3). TCL thickness yielded the highest AUC (0.945); at an optimal cutoff of 0.80 mm, sensitivity and specificity were 87% and 86%, respectively. CSA at the pisiform level (CSA1) showed the second-highest AUC (0.917), with 94% sensitivity and 75% specificity at a cutoff of 0.11 cm^2^. *Δ*CSA demonstrated a similar AUC (0.916), with both sensitivity and specificity of 85% at a cutoff of 0.05 cm^2^. SMI PR achieved an AUC of 0.893, corresponding to 79% sensitivity and 92% specificity at a cutoff of 2.05. SWE-derived elastic modulus (E) had an AUC of 0.887 (sensitivity 91%, specificity 75%) with a cutoff of 94.55 kPa, and shear wave velocity (SWV) showed a comparable AUC of 0.886 (sensitivity 87%, specificity 79%) with a cutoff of 5.64 m/s. CSA3 yielded an AUC of 0.839 (sensitivity 66%, specificity 86%) with a cutoff of 0.12 cm^2^, and FR2 yielded an AUC of 0.735 (sensitivity 70%, specificity 72%) with a cutoff of 3.97. CSA2 showed the lowest AUC (0.664), with 57% sensitivity and 76% specificity at a cutoff of 0.10 cm^2^.

**Table 3:** Area under the curve (AUC), optimal cutoff, sensitivity, and specificity of ultrasound parameters for CTS diagnosis.

Parameters	AUC (95% CI)	Cutoff value	Sensitivity (95% CI)	Specificity (95% CI)
CSA1	0.917 (0.869–0.965)	0.11 cm^2^	94% (0.825–0.987)	75% (0.644–0.829)
CSA2	0.664 (0.561–0.767)	0.10 cm^2^	57% (0.422–0.717)	76% (0.656–0.838)
CSA3	0.839 (0.770–0.908)	0.12 cm^2^	66% (0.507–0.791)	86% (0.775–0.924)
FR2	0.735 (0.647–0.823)	3.97	70% (0.551–0.827)	72% (0.622–0.811)
*Δ*CSA	0.916 (0.865–0.967)	0.05 cm^2^	85% (0.717–0.938)	85% (0.763–0.916)
TCL	0.945 (0.910–0.980)	0.80 mm	87% (0.743–0.952)	86% (0.775–0.924)
SMI PR	0.893 (0.827–0.959)	2.05	79% (0.643–0.893)	92% (0.839–0.963)
E	0.887 (0.831–0.942)	94.55 kPa	91% (0.796–0.976)	75% (0.644–0.829)
SWV	0.886 (0.831–0.941)	5.64 m/s	87% (0.743–0.952)	79% (0.691–0.865)

AUC, area under the receiver operating characteristic curve; CI, confidence interval.

CSA1–CSA4, median nerve cross-sectional area measured at the pisiform (carpal tunnel inlet), hook of hamate (carpal tunnel outlet), distal wrist crease, and pronator quadratus levels, respectively. FR1–FR4, flattening ratio measured at the corresponding levels; *Δ*CSA, difference in CSA between the pisiform and pronator quadratus levels; TCL, transverse carpal ligament thickness; SMI PR, superb microvascular imaging pixel ratio; E, elastic modulus; SWV, shear wave velocity.

### 3.4 Individualized BSA-based parameters for CTS diagnosis

**Figure 3:**
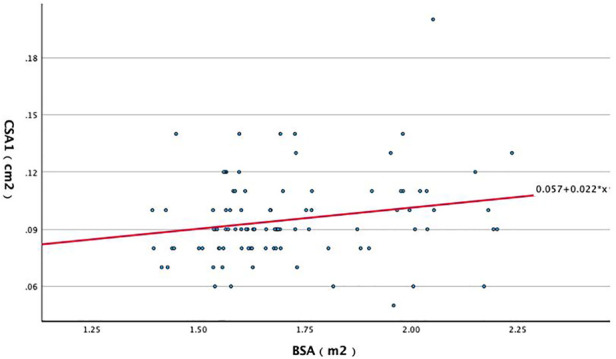
Scatter plot of the linear regression between BSA and median nerve CSA at the pisiform level.

A significant positive correlation was observed between body surface area (BSA) and CSA at the pisiform level (CSA1) (*R* = 0.230, *P* = 0.026). Linear regression analysis yielded a BSA-based prediction equation: *CSA1* *=* *0.022* *×* *BSA + 0.057* (Figure 3). This equation was used to derive individualized expected CSA1 values based on BSA.

**Figure 4:**
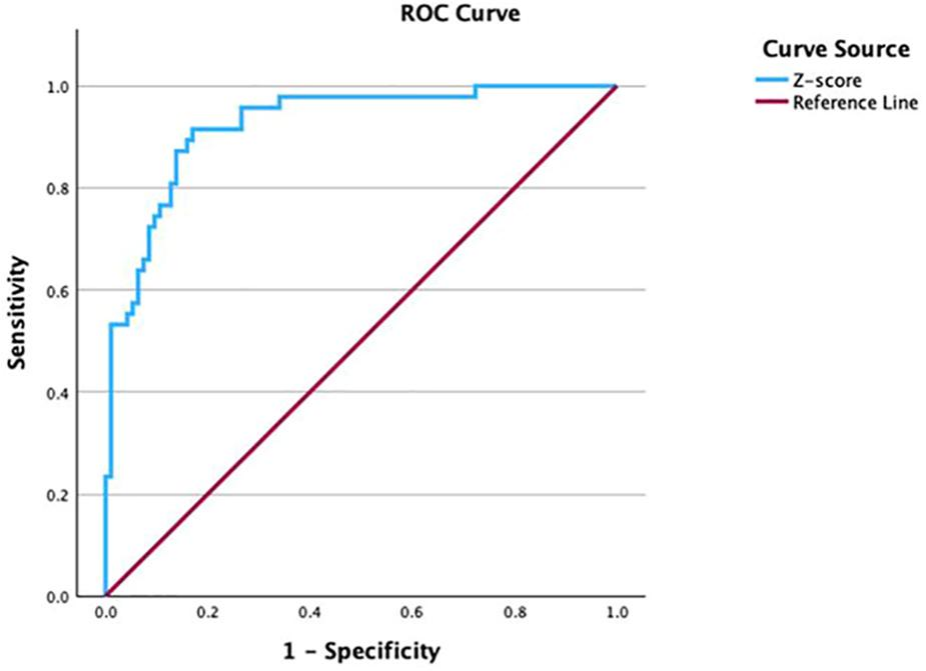
ROC curve of the BSA-based Z-score for CTS diagnosis.

Individualized Z-scores were calculated as: *Z* *=* *(X−μ)/σ*, where *X* denotes the mean of three repeated CSA1 measurements, μ is the BSA-predicted CSA1 from the regression equation, and σ is the standard deviation used for standardization. ROC analysis of the Z-score (Figure 4) demonstrated an AUC of 0.924 (95% CI 0.879–0.969), with an optimal cutoff of 0.7023, yielding 92% sensitivity (95% CI 0.796–0.976) and 83% specificity (95% CI 0.738–0.899).

To assess the clinical utility of this approach, McNemar's test was used to compare the classification performance of the BSA-based Z-score with the conventional fixed CSA1 threshold of 0.11 cm^2^. A significant difference was observed (*P* = 0.039). Compared with the fixed cutoff, the Z-score approach improved specificity (83% vs. 75%) while achieving comparable sensitivity (92% vs. 94%). These findings suggest that BSA-based individualized Z-scores may provide a more tailored diagnostic criterion for CTS, particularly by improving specificity.

### 3.5 Application of SMI and SWE parameters in CTS diagnosis

The diagnostic utility of Superb Microvascular Imaging pixel ratio (SMI PR) and shear wave elastography (SWE)–derived elastic modulus (E) was further evaluated using logistic regression analysis. CTS status was entered as the dependent variable, with SMI PR and E as independent predictors. Based on the estimated regression coefficients, a predictive model was established. Both SMI PR (*β* = 1.302) and E (*β* = 0.069) were independently associated with CTS (both *P* < 0.001; Table 4).

**Table 4:** Binary logistic regression analysis of SMI PR and elastic modulus for CTS diagnosis.

Predictors	β coefficient	Standard error (SE)	Wald *χ*^2^	df	Odds ratio (OR)	*P*-value
SMI PR	1.302	0.275	22.501	1	3.678	<0.001*
Elastic modulus (E)	0.069	0.017	17.083	1	1.072	<0.001*
Constant	−10.079	1.877	28.838	1		<0.001*

Binary logistic regression model with CTS diagnosis as the dependent variable and SMI PR and elastic modulus (E, kPa) as independent variables.

SMI PR, superb microvascular imaging pixel ratio; E, shear wave elastic modulus.

* *P* < 0.05 indicates statistical significance.

In the primary multivariable model, SMI PR (OR 3.678, *P* < 0.001) and elastic modulus (OR 1.072, *P* < 0.001) were independently associated with CTS. After additional adjustment for age and sex, SMI (OR 3.793, 95% CI 2.186–6.583; *P* < 0.001) and elastic modulus (OR 1.074, 95% CI 1.038–1.111; *P* < 0.001) remained significant, whereas age (OR 1.020, 95% CI 0.945–1.100; *P* = 0.620) and sex (male vs. female; OR 1.616, 95% CI 0.435–5.996; *P* = 0.473) were not statistically significant. The effect estimates changed minimally after adjustment (SMI: 3.678 vs. 3.793; elastic modulus: 1.072 vs. 1.074), indicating robustness to demographic confounding (Supplementary Table S1).

**Figure 5:**
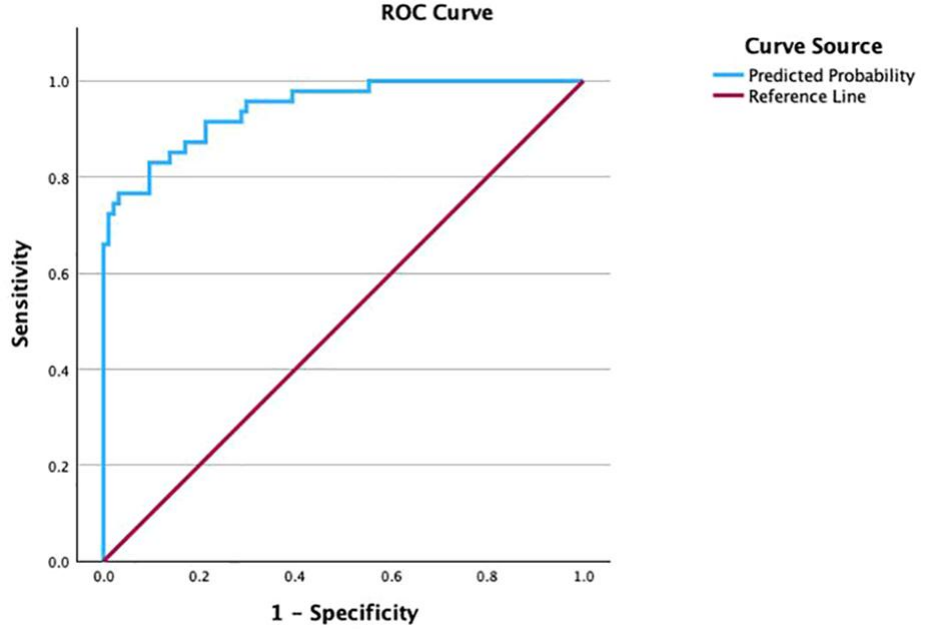
ROC curve of the combined logistic regression model using SMI and SWE for CTS diagnosis.

ROC analysis of the combined logistic regression model (Figure 5) showed an AUC of 0.944 (95% CI 0.906–0.982). At the optimal cutoff determined by the maximum Youden index, the model achieved a sensitivity of 83% (95% CI 0.692–0.924) and a specificity of 90% (95% CI 0.826–0.955), indicating high diagnostic performance for differentiating CTS wrists from controls.

These results suggest that integrating SMI PR and SWE-derived elastic modulus provides improved diagnostic accuracy compared with single-parameter assessment and may serve as a useful adjunct to conventional ultrasound evaluation for CTS.

## 4 Discussion

Carpal tunnel syndrome (CTS) is a common peripheral nerve entrapment disorder, primarily associated with pathological changes such as transverse carpal ligament thickening, flexor tendon fibrosis, and narrowing of the carpal tunnel (26, 27). Although electrophysiological examination (EDX) remains the diagnostic gold standard, it does not directly visualize structural alterations of the median nerve or surrounding tissues, which limits comprehensive assessment (28). With recent advances in high-frequency ultrasound and multimodal imaging, ultrasound has become increasingly important for CTS diagnosis by providing direct visualization of relevant anatomy and enabling quantitative evaluation of nerve morphology, intraneural vascularity, and mechanical stiffness.

This study showed that, among median nerve CSA–based metrics, CSA at the pisiform level (CSA1) provided strong diagnostic performance (AUC = 0.917), with 94% sensitivity and 75% specificity at an optimal cutoff of 0.11 cm^2^, consistent with prior reports (29–31). In addition, CSA2, CSA3, and *Δ*CSA also demonstrated useful discriminative ability, supporting the value of multi-level measurements for improving diagnostic assessment. However, a universally accepted CSA threshold has not been established, because inter-individual variability (e.g., sex, body habitus, and ethnicity) can substantially influence CSA measurements (9). To reduce the impact of such variability, several relative indices—such as the flattening ratio (FR), *Δ*CSA, and the CSA ratio (RCSA)—have been proposed (32). In the present study, *Δ*CSA achieved diagnostic performance comparable to CSA1 (AUC = 0.916), suggesting a potential advantage in partially accounting for individual differences.

Traditionally, CSA at the pisiform level (CSA1) has been applied using fixed cutoffs (e.g., 0.11 cm^2^) for CTS diagnosis in many studies. However, substantial inter-individual variation in body size may compromise diagnostic accuracy when a single threshold is used for all patients, potentially leading to false-positive or false-negative classifications, particularly at the extremes of stature (33). Although body mass index (BMI) is commonly used to characterize obesity, it has important limitations. First, BMI is derived solely from the weight-to-height ratio and therefore does not account for body composition, fat distribution, or muscle mass; for instance, individuals with high muscle mass may have BMI values similar to those with obesity despite markedly different body habitus. Second, BMI may correlate only weakly with the physiological dimensions of anatomical structures such as peripheral nerves, which may limit its utility for adjusting median nerve CSA and could contribute to diagnostic imprecision.

To address these limitations, we evaluated body surface area (BSA) as an anthropometric adjustment for CSA-based CTS assessment. BSA, calculated using the Du Bois formula from height and weight, provides an integrated measure of body size and may better reflect the physiological dimensions of peripheral nerves and musculoskeletal structures than BMI. By accounting for body-size variability, BSA-based adjustment may reduce misclassification when a single fixed CSA threshold is applied across individuals. Using linear regression, we established a BSA-based prediction equation for CSA1: *CSA = 0.022* *×* *BSA + 0.057*. We then derived individualized BSA-based Z-scores to optimize the diagnostic application of CSA1. The Z-score model achieved an AUC of 0.924 with 92% sensitivity and 83% specificity, compared with the conventional fixed cutoff of 0.11 cm^2^ (AUC 0.917; sensitivity 94%; specificity 75%). McNemar's test demonstrated a significant difference between these approaches, indicating that the BSA-based Z-score improved classification performance, particularly by enhancing specificity. Collectively, these findings support the use of BSA-based individualized criteria to improve the accuracy and generalizability of CSA-based ultrasound diagnosis for CTS, offering a more personalized alternative to fixed thresholds.

The flattening ratio (FR) reflects morphological deformation of the median nerve associated with compression, and several studies have proposed FR as an adjunctive parameter for CTS diagnosis (34). However, FR may be strongly affected by inter-individual anatomical variation and measurement conditions, which can compromise its stability and reproducibility. To date, no standardized diagnostic cutoff has been established, and reported sensitivities vary widely (approximately 38%–65%) (35, 36). In our study, only FR at the hook of hamate level (FR2) differed significantly between CTS wrists and controls, yielding an AUC of 0.735 with 70% sensitivity and 72% specificity at a cutoff of 3.97. Overall, FR2 demonstrated lower diagnostic performance than CSA1 and *Δ*CSA, suggesting that the role of FR as an auxiliary indicator warrants further validation.

The transverse carpal ligament (TCL) is a key anatomical component of the carpal tunnel, and TCL thickening has been implicated in CTS pathophysiology by reducing tunnel space and increasing compressive stress on the median nerve. Prior studies have reported an association between TCL thickness and CTS occurrence as well as postoperative outcomes (37). In the present study, TCL thickness yielded the highest diagnostic performance (AUC = 0.945), with 87% sensitivity and 86% specificity at an optimal cutoff of 0.80 mm, supporting its potential value as a diagnostic marker. Nevertheless, further studies with larger and more diverse populations are warranted to confirm the robustness and generalizability of TCL measurements, given potential variability related to ultrasound equipment, operator experience, and measurement protocols.

Shear wave elastography (SWE) enables quantitative assessment of tissue stiffness using minimal probe pressure and provides objective biomechanical information, with advantages over conventional strain elastography (38, 39). Chronic median nerve compression in CTS has been associated with fibrotic and sclerotic changes, which may increase nerve stiffness. In the present study, the SWE-derived elastic modulus (E) demonstrated good diagnostic performance (AUC = 0.887), yielding 91% sensitivity and 75% specificity. Shear wave velocity (SWV) showed comparable performance (AUC = 0.886), with 87% sensitivity and 79% specificity.

Superb Microvascular Imaging (SMI) enables sensitive visualization of slow and low-volume intraneural blood flow. Previous studies have reported increased microvascular density in CTS, potentially related to Schwann cell activation and neuroinflammatory responses (40). In the present study, SMI PR achieved an AUC of 0.893 with high specificity (92%), supporting the value of SMI for detecting abnormal intraneural perfusion. Given its technical capability to depict low-velocity flow, SMI may provide improved sensitivity compared with conventional color and power Doppler ultrasound (CDUS/PDUS), as suggested in prior reports (41).

Furthermore, the combined model incorporating SMI PR and elastic modulus (E) improved overall diagnostic performance, yielding an AUC of 0.944 with 83% sensitivity and 90% specificity at the optimal threshold (maximum Youden index = 0.734). Notably, the combined approach preserved high sensitivity while achieving higher specificity, which may reduce false-positive classifications and supports the robustness of integrating complementary imaging biomarkers rather than relying on a single parameter. Our findings are consistent with Karahan et al., who reported an association between SMI findings and CTS severity (42), and with recent studies highlighting the diagnostic value of SWE (43). Collectively, SMI and SWE provided meaningful diagnostic information individually, and their combined model further improved diagnostic efficiency and potential clinical applicability.

Despite the potential clinical value of the individualized BSA-based Z-score and the combined SMI–SWE model, several limitations should be acknowledged. First, the sample size was relatively small. Second, ultrasound is inherently operator-dependent. This concern may be more pronounced for newer techniques such as SMI and SWE, for which acquisition settings and quantification protocols have not been fully standardized, potentially affecting reproducibility. Third, electrophysiological examinations were not performed in the control group; therefore, subclinical neuropathies could not be completely excluded, which may have influenced specificity estimates.

High-frequency ultrasound combined with Superb Microvascular Imaging (SMI) and Shear Wave Elastography (SWE) provides valuable diagnostic information for carpal tunnel syndrome (CTS). Among two-dimensional parameters, CSA at the pisiform level (CSA1), transverse carpal ligament (TCL) thickness, and *Δ*CSA showed strong diagnostic performance. A body surface area (BSA)–based individualized Z-score improved the specificity of CSA compared with a fixed cutoff, and the combined SMI–SWE model further enhanced overall diagnostic performance. Larger multicenter studies are needed to confirm generalizability and to define the role of multimodal ultrasound in early diagnosis and follow-up of CTS (44).

## Data Availability

The raw data supporting the conclusions of this article will be made available by the authors, without undue reservation.
