# Supplementary material for: Body surface area–adjusted median nerve cross-sectional area and multimodal ultrasound improve diagnosis of carpal tunnel syndrome
Source: Front Surg. 2026 Feb 23;13:1774737. doi: 10.3389/fsurg.2026.1774737 (PMC12967954; doi:10.3389/fsurg.2026.1774737)
Supplement: Supplementary file 1 [file Datasheet1.pdf]

Supplementary Table S1. Binary Logistic Regression Analysis additionally adjusted for age and sex

	β Coefficient	Standard Error (SE)	Wald χ^2	df	Odds Ratio (OR)	P-value
SMI PR	1.333	0.281	22.472	1	3.793 (2.186–6.583)	<0.001*
Elastic modulus(E)	0.071	0.017	16.777	1	1.074 (1.038–1.111)	<0.001*
Age	0.019	0.039	0.246	1	1.020 (0.945–1.100)	0.620
Sex	0.480	0.669	0.515	1	1.616 (0.435–5.996)	0.473
Constant	-11.511	3.284	12.287	1		<0.001*

Note: Binary logistic regression model with CTS diagnosis as the dependent variable and SMI PR, elastic modulus, age, and sex as independent variables. Sex was coded as male = 1 and female = 0 (reference = female). OR values are presented with 95% confidence intervals in parentheses.

*$P < 0.05$ indicates statistical significance.
